# Whole exome sequencing identified a new compound heterozygous *PRKN* mutation in a Chinese family with early-onset Parkinson’s disease

**DOI:** 10.1042/BSR20200534

**Published:** 2020-05-20

**Authors:** Tianbai Li, Daqing Kou, Yanhua Cui, Weidong Le

**Affiliations:** 1 Center for Clinical Research on Neurological Diseases, The First Affiliated Hospital, Dalian Medical University, Dalian 116021, China; 2 Liaoning Provincial Key Laboratory for Research on the Pathogenic Mechanisms of Neurological Diseases, The First Affiliated Hospital, Dalian Medical University, Dalian 116021, China; 3 Department of Clinical Laboratory, The First Affiliated Hospital, Dalian Medical University, Dalian 116021, China; 4 International Education College, Dalian Medical University, Dalian 116044, China

**Keywords:** dopa-responsive dystonia, Early-onset Parkinson’s disease, Mutation, PRKN, Whole exome sequencing

## Abstract

Early-onset Parkinson’s disease (EOPD) is usually caused by genetic variants and patients with EOPD develop symptoms before the age of 50, accounting for 5% Parkinson’s disease (PD). Here we present a Chinese Han pedigree with clinical features of EOPD. To determine the diagnosis and pathogenic mutations of this pedigree, whole exome sequencing, Sanger sequencing and real-time quantitative PCR were performed to detect all the four family members. Our results showed that a new form of compound heterozygous mutation in the *PRKN* gene, consisting of heterozygous point mutation c.850G > C (p.G284R) along with exon 4 deletion, is the causative genetic factor for EOPD in this pedigree. These discoveries may have implications for genetic counseling, clinical management and developing *PRKN* target gene therapy strategy.

## Introduction

Parkinson’s disease (PD) is a progressive neurodegenerative disorder that results primarily from the death of dopaminergic neurons in the substantia nigra [1,2]. As the second most frequent neurodegenerative disease after Alzheimer’s disease, PD typically occurs in people over the age of 60, of which approximately 1% are affected [3,4]. When it is seen in patients before the age of 50, it is called early-onset PD (EOPD), accounting for 5% of PD [5–7]. The characteristic features of EOPD have been demonstrated to be a better response to dopaminergic drugs, more frequently gait disorder, rest tremor of the legs and limb dystonia comparing with the patients with older age of onset, but cognitive decline and hyposmia are relatively uncommon [5,8].

Although most cases of PD are thought to be sporadic, 5–10% of patients are confirmed to have monogenic forms of the disease [9]. Susceptibility genes for Mendelian-inherited PD have been reported, including *LRRK2*, *SNCA*, *PRKN* (*parkin*), *PINK1*, *GBA*, *PARK7*, *UCHL1*, *ATP13A2*, *VPS35*, *NR4A2* and so on [4,10–12]. The role of pathogenic variants is more conspicuous in EOPD [13]. Among the loss-of-function mutations which cause recessive EOPD such as *PRKN*, *PINK1*, *DJ-1* and *ATP13A2* [8,9], homozygous and compound heterozygous mutations in the *PRKN* gene are responsible for approximately half of recessive-inheritance PD families with onset before the age of 45 years [6].

On account of the phenotypes of EOPD are associated with the age of onset and genotypes, and different genetic backgrounds could generate diverse pathogenesis, a comprehensive family-based genetic testing in EOPD was revealed to be required. Whole exome sequencing (WES) has growingly been utilized as a clinical diagnostic tool of EOPD in recent years, for its advantage in identifying abundant gene mutations and differential diagnosis with other dopa-responsive disorders such as dopa-responsive dystonia (DRD) [14–17]. In the present study, we present a Chinese Han pedigree with clinical features of EOPD and finally confirmed the diagnosis with a new form of compound heterozygous mutation in the *PRKN* gene, consisting of point mutation c.850G > C (p.G284R) along with exon 4 deletion (EX 4 del) using the WES method. This finding may enrich the broad spectrum of genotypes in the EOPD and have implications for genetic counseling.

## Methods

### Subjects

**Figure 1:**
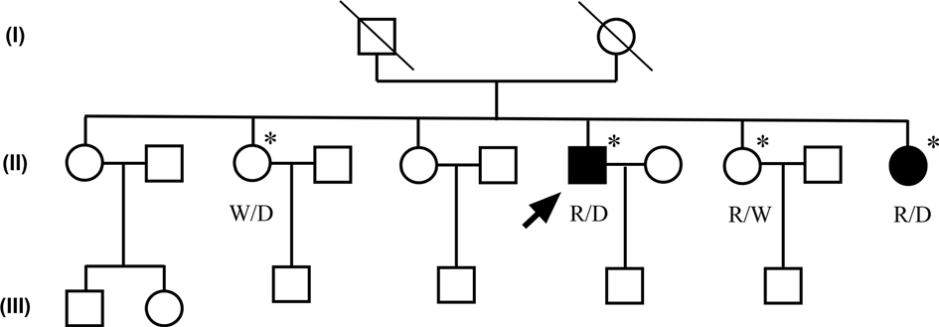
The EOPD pedigree with the compound heterozygous mutations in the *PRKN* gene Pedigree of EOPD with the compound heterozygous mutations of the *PRKN* gene (p. G284R and EX 4 del) showing affected cases (fully shaded). The arrow indicates the proband with EOPD. *Individuals included in the WES. R, the allele for the p. G284R mutation; D, the allele for the EX 4 del mutation; W, wild-type allele; Circles, women; Squares, men.

All members of the family recruited in the present study were of Chinese Han descent, who were born in Dalian, China. The kindred comprised three generations, of which two second-generation family members (II: 4, II: 6) were affected (Figure 1). Peripheral blood of four available family members (II: 2, II: 4, II: 5 and II: 6) was sampled for the genetic analysis. All participants had neurological examinations performed. Informed written consent was obtained from all participating subjects in the present study. Ethical approval was obtained from the Ethics Committee of the First Affiliated Hospital of Dalian Medical University (approval number: LCKY2014-29).

###  WES

A total of 2 ml peripheral venous blood from the four family members and two healthy controls was drawn into EDTA-anticoagulant tubes. gDNA was extracted from peripheral blood using TaKaRa MiniBEST Whole Blood Genomic DNA Extraction Kit (Takara, Dalian, China). The gDNA samples were submitted to YiZi Corporation (Yizi, Shanghai, China) for WES. Briefly, 1.6 μg of gDNA was sheared into fragments with the Covaris E220. DNA libraries were constructed and the whole exome capture was performed using the Illumina TruSeq™ (Illumina Inc) Exome Enrichment Kit. Massively parallel sequencing was executed by utilizing the Illumina HiSeq2000 platform and version 3 SBS chemistry to generate 100-bp paired-end reads.

### Genomic analysis

Genomic analysis of all the subjects in the present study was carried out by Smartquerier Biotechnology Institute (Shanghai, China). An acceptable quality of sequencing data were substantiated by using FastQC (Version 0.11.2). All reads were aligned using Burrows–Wheeler Aligner against the UCSC hg19 reference genome. SpeedSeq as an open-source genome analysis platform that accomplishes alignment, variant detection of a 50× human genome [18], was utilized to detect single-nucleotide variants (SNPs), structural variants, insertions, deletions and so on. By further using ANNOVAR to annotate the variants and conservative predictions. To predict the pathogenesis of the variants, both of the wild and mutant gDNA sequences were analyzed using the Human Gene Mutation Database (HGMD, http://www.hgmd.cf.ac.uk/docs) and ClinVar (https://www.clinicalgenome.org/data-sharing/clinvar/). Sanger sequencing was then performed in family members and two healthy controls to confirm the identified potential pathogenic variants. PCR amplification and Sanger sequencing primer sequences are as follows: 5′-CCACGTGATTTGCTTAG-3′ and 5′-TTTCATATCCAGCAATGATCAAA-3′. For the validation of EX 4 del in the *PRKN* gene, we performed gDNA real-time quantitative (RT-qPCR) by using ABI 7500 fast real-time PCR system (Applied Biosystems, Foster City, CA, United States) in a total volume of 20 µl for each reaction. A specific PCR primer pair (5′-GAGTTTCTTGTCTCAATTTAGATGC-3′ and 5′-TTTCTTTTCAAAGACGGGTGA-3′) was used for amplification of a 122-base pair fragment in exon 4 of *PRKN*. The *GAPDH* gene was amplified as a housekeeping gene. After 94°C for 30 s, the experimental reaction consisted of 40 cycles of 94°C for 5 s and 60°C for 34 s by the fluorescent dye SYBR Green I Kit (TransGen, Beijing, China). The value of threshold cycle (*C*_t_) was generated at every cycle during a run. The dose of the *PRKN* exon 4 relative to GAPDH was determined using the 2^−△△*C*_t_^ method. Gene dosage alternations were confirmed after triple analysis.

## Results

### Clinical history

The index case (II: 4) in this pedigree was a 62-year-old man born to healthy, non-consanguineous parents. His disease began at the age of 24 as uncoordinated movements in his left leg, leading to cycling instability. During the 2 years after the disease onset, his postural instabilities were slowly progressing to his right leg and arms, which manifested as festinating and abnormal stiff-legged gait, easy to fall and poor handwriting. The above symptoms were typically absent in the morning but worsened during the day. He had mild bradykinesia, no tremor, muscular atrophy or cognitive impairment. There were no indications of sphincteric or other autonomic symptoms. At the age of 30, he started pharmacological treatment with a daily dosage of 100 mg of levodopa showing a good response. Levodopa-induced dyskinesia (LID) appeared 5 years after the commencement of drug therapy. Neurological examinations showed increased muscular tension, hyperreflexia and Babinski sign in his left leg, other neurological and neuropsychiatric examinations indicated no abnormal results. An MRI brain scan revealed no significant abnormality. Video showing patient II: 4 is available in Supplementary Video S1.

Patient II: 6, who is the younger sister of II: 4, was examined at the age of 53. When she was 24 years old, she manifested parkinsonian features including rest tremor, bradykinesia, postural instability and hypomimia. She also had hyperreflexia and bilateral Babinski sign. After being treated with levodopa for the past 30 years, her symptoms have improved, and the LID has not appeared (Supplementary Video S1). Detailed clinical characteristics for the family are summarized in Table 1.

**Table 1:** Mutations in the *PRKN* gene and clinical data of the pedigree

Subject	II:2	II:4	II:5	II:6
Age (yr)	67	62	58	53
Sex	M	F	M	M
Genotype	Het	Com Het	Het	Com Het
*PRKN* p.G284R	-	Het	Het	Het
*PRKN* exon 4 deletion		Het	-	Het
Phenotype	Het	Affected		Affected
Age at onset (yr)	Carrier	24	Carrier	24
	-		-	
Tremor	-	-	-	+
Rigidity	-	+	-	+
Bradykinesia	-	+	-	+
Gait disturbance	-	+	-	-
Diurnal fluctuations	-	+	-	-
Hyperreflexia	-	+	-	+
Effect of levodopa	-	+	-	+
LID	-	+	-	-
Dementia	-	-	-	-
Sleep disorders	-	-	-	-

Abbreviations: Com Het, compound heterozygote; F, female; Het, heterozygote; M, male; yr, year.

### Genetic analysis

**Figure 2:**
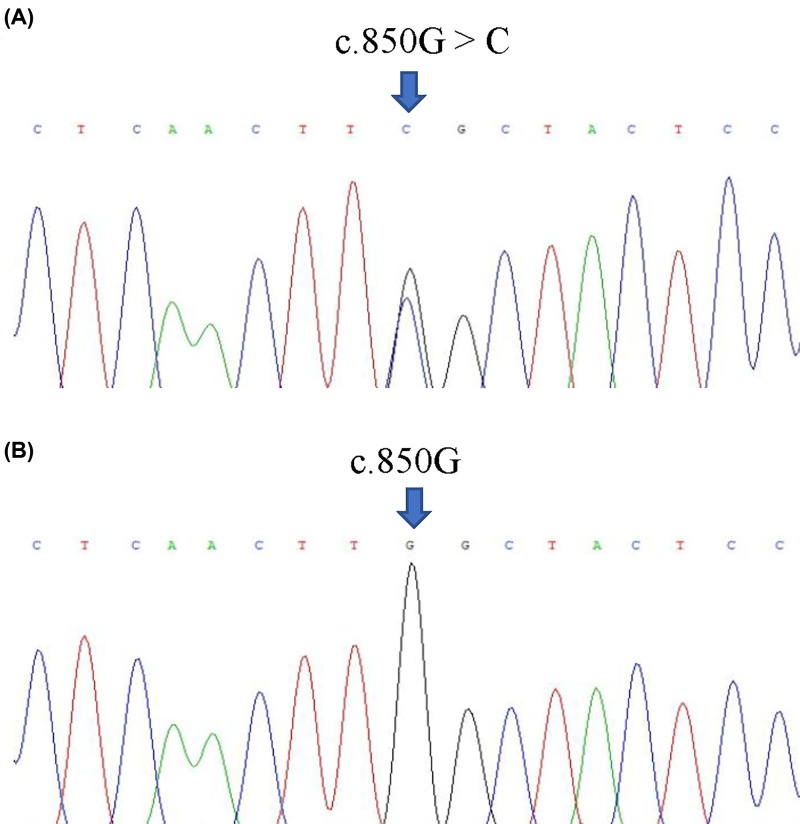
Sanger sequencing of the c.850G > C variant in the *PRKN* gene (**A**) The sequence of heterozygous c.850G > C variant in II: 4, II: 5 and II: 6. (**B**) The sequence of II: 4 and healthy control.

WES was performed on four family members and two unrelated healthy controls. Average exome sequencing depths of 95.11× (II: 2), 100.65× (II: 4), 94.33× (II: 5) and 94.73× (II: 6) provided sufficient accuracies to call variants in 99.85–99.89% of the target regions. After filtering known variants and performing functional predictions, a total of 50944 SNPs, 2435 insertions and 2681 deletions in the exon regions were identified. We found that heterozygous *PRKN*: c.850G > C (p.G284R) variant was observed in II: 4, II: 5 and II: 6. Furthermore, heterozygous p.G284R variant was confirmed by Sanger sequencing (Figure 2A), and this variant was absent from unaffected family member II: 2 and healthy controls (Figure 2B). The p.G284R variant (NP 004553.2) was recorded in the NCBI SNP database (rs751037529). MutationTaster predicted that the variant has a high possibility of disease-causing with a high probability near 1. The conservation analysis showed that glycine at position 284 (p.G284) is highly conserved among various vertebrates based on multiple sequence alignments [19], which suggests that p.G284R might be a pathogenic mutation.

**Figure 3:**
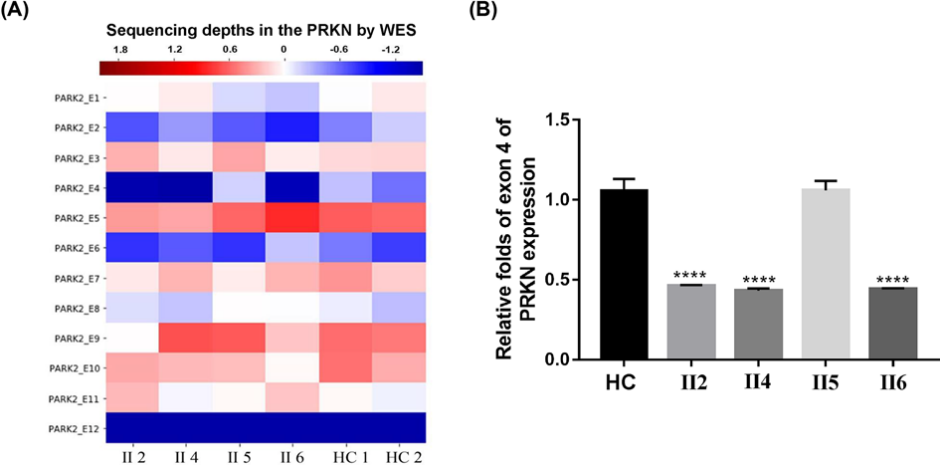
Exon deletion analyses using WES and RT-qPCR (**A**) The sequencing depths analysis for PRKN exons of the pedigree and healthy controls (HC). The color-axis represents the sequencing depths performed by WES. The sequencing depths of samples II: 2, II: 4 and II: 6 in the *PRKN* exon 4 (PARK2_E4) was relatively lower than that of healthy controls (HC1 and HC2). (**B**) RT-qPCR analysis for exon 4 of PRKN showed the relative expression level of exon 4 in PRKN is decreased in II: 2, II: 4 and II: 6 than that in II: 5 and HC. ****P<0.0001.

Then exon deletions were evaluated through the sequencing depths analysis in the *PRKN* gene, heterozygous *PRKN* exon 4 del was detected in II: 2, II: 4 and II: 6 (Figure 3A). RT-qPCR analysis for exon 4 of *PRKN* indicated that the dose of exon 4 in *PRKN* is decreased in the II: 2, II: 4 and II: 6 than that in II: 5 and healthy controls (Figure 3B), and the outcome obtained were consistent with the sequencing depths analysis. These results indicated that patients II: 4 and II:6 have compound heterozygous mutations in the *PRKN* gene (Table 1). The two other family members (II: 2 and II:5) lacking EOPD phenotypes only carry the single heterozygous mutation, identifying them as a carrier of the *PRKN* mutation. Except for the p.G284R variant and EX 4 del in the *PRKN* gene, no other variants in known PD disease-causing genes were found in the family members. We, therefore, identified the genetic cause of this family with EOPD.

## Discussion

Although the patients in the present study presented with typical symptoms of *PRKN*-type EOPD, including onset at an early age, beneficial response to levodopa, diurnal fluctuations and the occurrence of levodopa-related motor complications. Other dopa-responsive disorders such as DRD should not be excluded during the diagnosis according to the clinical manifestations. Therefore, in the present study, we performed family-based genetic testing by using WES and finally identified a compound heterozygous mutation (p.G284R and EX 4 del) in the *PRKN* gene from a Chinese family with EOPD. In this family, two of four siblings of unaffected parents developed PD, indicating an autosomal recessive pattern of inheritance. The p.G284R variant was recorded in the SNP database with a frequency of <0.001%, and to our knowledge, it is the first report of these mutations in compound heterozygotes.

The *PRKN* gene is a large 1.3 Mb gene with 12 exons that are mapped to chromosome 6q34 and encodes an E3 ubiquitin-proteasome system [20]. Mutations of the *PRKN* gene were first identified in several patients with autosomal recessive EOPD from consanguineous families in Japan [21]. Since then more than 200 different mutations in the *PRKN* gene have been identified in homozygous or compound heterozygous state of EOPD patients, including point mutations, complex rearrangements (exon deletions and multiplications) [22,23]. The G284 codon lies in exon 7 of the *PRKN* gene (NM_004562). Although the *PRKN* p.G284R variant was included in the NCBI SNP database (rs751037529) and several studies have also reported the point mutation of p.G284R contributes to the development of EOPD [24–26], the frequency of this variant is very low (<0.001%). And more notably, the cases who have been reported to have p.G284R mutation in the *PRKN* gene, to our knowledge, are all Chinese descent. We suspect that *PRKN* p.G284R mutation could be more common in Chinese patients.

In the present study, all carriers with single heterozygous mutation (p.G284R or EX 4 del) were free of any neurological symptoms, which support the view that one heterozygous mutation in the *PRKN* gene was insufficient and a second mutation was required in EOPD. A recent study screened the phenotypic and genotypic data in more than 1100 patients with recessively inherited EOPD from a total of 3652 citations by using MDS Gene’s standardized data extraction protocol and found that 53.2% of the patients carried a homozygous mutation, and 43.6% were compound heterozygous in a total of 139 different disease-causing sequence variants reported in the *PRKN* gene [5]. Mounting evidence indicated that a single *PRKN* mutation might be a risk factor for the late-onset disease [27,28]. Therefore, follow-up of the heterozygous mutation carriers is suggested to investigate their clinical features.

*PRKN*-type EOPD always shares similar clinical features to patients with DRD. The difficulty in distinguishing between those two forms of dopa-responsive disorders has already been widely reported [14,15]. It is suggested that a genetic mutation characteristic for DRD should also be included when testing for the causative genes in patients with EOPD. Strategies based on WES are very attractive because of their high-efficiency and universality of application, which means that WES is applicable for whatever the group of disorders and the clinical features [29]. It could be widely used in the future, for it could improve clinical diagnosis and genetic counseling of EOPD.

In conclusion, this report describes a new compound heterozygous mutation (p.G284R and EX 4 del) in the *PRKN* gene from a family with EOPD by performing WES. This finding may enrich the broad spectrum of genotypes in the EOPD and have implications for genetic counseling. This case also emphasizes the significance of WES in the diagnosis of EOPD. As prices drop, WES could be widely used in the future for its high-efficiency and full-coverage, which may help improve the clinical diagnosis and genetic counseling of EOPD.

## Supplementary Material

Supplementary Video S1

## Abbreviations

DRD: dopa-responsive dystonia
EOPD: early-onset Parkinson’s disease
EX 4 del: exon 4 deletion
LID: levodopa-induced dyskinesia
PD: Parkinson’s disease
SNPs: single nucleotide polymorphisms
WES: whole exome sequencing
